# Development and adaptation of patient-reported outcome measures for patients who experience itch associated with primary biliary cholangitis

**DOI:** 10.1186/s41687-018-0090-1

**Published:** 2019-01-15

**Authors:** Mona L. Martin, Larissa Stassek, Steven I. Blum, Ashish V. Joshi, David Jones

**Affiliations:** 1 Health Research Associates, Inc., 6505 216th St. SW, Suite 105, Mountlake Terrace, WA 98043 USA; 2 GlaxoSmithKline, 1250 S. Collegeville Road, Collegeville, PA 19426 USA; 3 Newcastle University, Newcastle upon Tyne, UK

**Keywords:** Itch, Patient-reported outcomes, Primary biliary cholangitis, PBC, Pruritus, Qualitative

## Abstract

**Purpose:**

To conduct qualitative interviews to evaluate and refine the Itch Diary (ID) and weekly version of the PBC-40 in patients with itching associated with primary biliary cholangitis (PBC).

**Methods:**

Twenty adults with self-reported PBC diagnoses and recent/ongoing itching of at least moderate intensity participated in face-to-face qualitative combined concept elicitation (CE) interviews and cognitive interviews after completing the morning and evening versions of the ID and weekly version of the PBC-40. These questionnaires were evaluated to confirm saturation of concepts of interest and cognitively test the English language versions of the measures in patients with PBC in the US and Canada. Transcripts were organized into descriptions of PBC-related symptoms and symptom-related impacts using a structured coding framework. Two waves of interviews were conducted; revisions made after wave 1 were further tested in wave 2.

**Results:**

Interview results confirmed the relevance of concepts presented in the PBC-40 and ID to patients’ experiences. Saturation of concept was achieved. Itching-related signs and symptoms (46%) were the most commonly expressed symptom concept in the CE interviews followed by energy-related (14%) and additional signs/symptoms (13%). Several changes to the ID were made in response to cognitive interview results. Changes to the PBC-40 included adaptations from British to North American English, and the appropriateness of a 7-day recall period was confirmed.

**Conclusions:**

Relevance of the symptom and impact concepts in the ID to measure PBC-related itch were confirmed. Adaptation of the PBC-40 to a weekly recall period and for North American English was successful.

## Introduction

Primary biliary cholangitis (PBC), previously known as primary biliary cirrhosis, is a chronic autoimmune disease of the liver in which intrahepatic small bile ducts are selectively destroyed by inflammatory cells [1, 2]. The disease is progressive and eventually results in liver fibrosis [2]. PBC is relatively uncommon, with a prevalence of up to 39.2 per 100,000 in the United States [3], and has a strong predominance in women [3, 4].

Itching (pruritus) is an extrahepatic symptom of PBC that occurs in an estimated 55% of patients [5]. The itch may be severe and can have a significant impact on patients’ health-related quality of life [6]. In a survey of 238 patients with PBC, the itching sensation was described as “bugs crawling” or “relentless” and 3.6% reported they scratched until they bled [7]. Nearly three-quarters of patients reported that the itch interfered with sleep [7]. Scratching brings little to no relief and intense scratching may cause serious damage to the skin [8]. The bile acid sequestrant, cholestyramine is the only US Food and Drug Administration (FDA)-approved treatment for PBC-related itch, but it has an unpleasant taste and can cause gastrointestinal side effects, limiting its clinical uptake. Additionally, it should not be taken within 4 to 6 h of many other common medications (e.g., warfarin, digoxin, seizure medications, levothyroxine, antibiotics, etc) [1, 8].

Itch is a sensation that cannot be directly observed, and is only known to the individual, making its assessment subjective and difficult to quantify [8]. Patient-reported outcome (PRO) measures for PBC-related itch are limited. Tools used to date include a simple grading of worst itch over a certain period (e.g., the past 7 days) on a numeric rating scale (e.g., 0 to 10) or on a Visual Analogue Scale (VAS) and the 5-D itch scale [8]. The 5-D itch scale is a brief and easy-to-complete measure designed to specifically evaluate the extent and impact of itch across a range of conditions, but it was not developed using qualitative interviews with PBC patients [9]. On the other hand, the PBC-40 is a PBC-specific, health-related quality-of-life questionnaire, but it only has 3 questions to assess itch and these questions may not be sufficient to cover the spectrum of itching, which is a key symptom in this target population [10]. Additionally, the original version of the PBC-40 has a 4-week recall period, which may not be ideal for measuring daily variability in patient’s itch experience.

The Patient Reported Symptoms Questionnaire (“Itch Diary”) was developed to meet the need for a PBC-specific itch PRO measure and was based on key concepts in the PBC-40 [11, 12]. The questionnaire focuses on itch, but also contains items for a few other PBC symptoms such as fatigue and diarrhea. Initial cognitive testing was conducted through interviews with 10 participants with PBC, and the Itch Diary was preliminarily used in a phase 2a safety and tolerability study of GSK2330672, an ileal bile acid transporter inhibitor under development for PBC-related itch [13]. In order to ensure greater accuracy in the recall of symptoms, the phase 2a study also preliminarily evaluated the PBC-40 during a 2-week placebo run-in period using a “past 2 weeks” recall period, in contrast to the original recall period of “last 4 weeks” [10, 14]. However, it was still unclear as to whether the list of symptoms was complete in terms of key concepts relevant to those with PBC.

An integral part of developing quality-of-life measures is to conduct qualitative patient interviews and such interviews are recommended by the FDA for the development of PRO measures [15, 16]. The objective of the current study was to further develop and cognitively test English versions of the Itch Diary and the weekly version of the PBC-40 in North American individuals with PBC and related itching, and to identify any other potential concepts that might be relevant to patients’ experiences.

## Methods

### Study design and participants

While the qualitative sample of ten patients in the previous study at times may be acceptable for rare conditions, it was still unclear whether the list of symptoms identified in the earlier study was complete in terms of the key concepts relevant to people with PBC, and whether the content was transferrable to other countries and cultures. Therefore, we conducted additional qualitative interviews in patients with PBC to confirm saturation of the concepts of interest and the applicability of these concepts within the US and Canada. In-person qualitative interviews consisting of concept elicitation (CE) and cognitive portions were conducted in the US and Canada to collect data for confirming content relevance, instrument feasibility, and saturation of concept for the Itch Diary and the weekly version of the PBC-40. Interviews lasted for about 90 min, beginning with the CE portion of the interview, then completion of questionnaires of interest (PBC-40, Itch Diary) as well as some additional secondary questionnaires (Weekly Gastrointestinal Diary; Patient Global Impression of Severity for Itch [PGIS-Itch] and Patient Global Impression of Change for Itch [PGIC-Itch]; and 5-D itch scale), followed by the cognitive portion of the interview.

Men and women aged 18 to 80 years who self-reported a diagnosis of PBC were recruited via local and national patient groups. Participants were required to have ongoing itching of at least moderate intensity (equivalent to a score of ≥4 on the numerical response scale [NRS; range 0–10]) during the previous 8 weeks. Individuals with a self-reported history of liver disease of other etiology or reporting other highly symptomatic medical conditions (i.e., inflammatory bowel disease, chronic plaque psoriasis, eczema, etc) were excluded. As PBC predominantly affects women [3, 4], a majority of the sample was expected to be female.

Study recruitment was conducted via advertisements to members of patient groups in the US and Canada: PBCers and the Canadian PBC Society. The study was conducted in compliance with Health Insurance Portability and Accountability Act regulations, and all participants provided written informed consent. The study was approved by an Institutional Review Board (Quorum Review IRB, Seattle, WA, USA).

### Data collection

Demographic and basic comorbidity data were collected from each participant. Those who were willing, signed a Medical Information Release Waiver giving their consent for additional limited medical information to be obtained from their medical provider. Information collected from the medical provider, when authorized, included date of PBC diagnosis, current and past medications for itching/pruritus, previous history of liver transplant (yes/no), and, when possible, the most recent bilirubin and alkaline phosphatase lab results.

### Interview process

Interviewers were experienced in interviewing techniques for PRO measurement development and in conducting one-on-one interviews across a wide range of therapeutic areas. Training for the interviewers included an instruction session specific to the study, review of the interview guide, and observed practice interviews.

Interviews were conducted in 2 waves of 10 participants each. Revisions to the PRO measures were made in response to participant input from the first wave, and then tested in the second wave. During the CE portion of the interviews, participants were asked open-ended questions in order to elicit spontaneous reports of the patient’s experience, followed by probing questions designed to more fully explore participant experiences with PBC symptoms (including frequency, severity, and variation of symptoms) and the impact of PBC on emotions, daily activities, social functioning, sleep, etc. During the cognitive portion of the interviews, participants were asked a series of structured and semi-structured questions designed to obtain feedback on the PRO measures. Topics covered were the participants’ interpretation of the individual items, how they regarded the fit and adequacy of the response scales and recall periods, and how comfortable they were with the terminology used. Participants were also asked to discuss the relevance of PRO items and whether any other important concepts were missing and should be considered for addition.

PRO measures of interest were administered to each participant on paper between the CE and cognitive portions of the interview. The PROs of main interest were the Itch Diary (AM and PM versions) and a weekly version of the PBC-40 that used a 7-day recall period as opposed to the original 4-week recall period [10]. The PBC-40 contains 40 questions covering 6 domains (symptoms, itch, fatigue, cognition, social, and emotional), and 3 additional questions measuring general health status [10]. The PGIS-Itch and PGIC-Itch were also evaluated to confirm their relevance as suitable anchor measures for use in future studies to assess the psychometric properties of the Itch Diary and the weekly version of the PBC-40.

### Data analysis

Transcripts from the CE portion of the interview were organized into descriptions of PBC-related symptoms and symptom impacts by developing a coding framework and coding dictionary. Two coders were used during the study. Inter-rater agreement was evaluated by independent dual coding of 2 of the 20 transcripts and comparing the coding differences and expressing coding consistency by percentage of agreement in the codes assigned.

A saturation table was used to track symptoms and impacts from the concept elicitation portion of the interviews. The transcripts were ordered chronologically, based on interview completion date, and then grouped into four groups of five transcripts each. Concept saturation was then evaluated by comparing the codes in each new transcript group with the codes in the previous group to identify newly appearing information. When new codes no longer appeared, no new information was being obtained; this is called “saturation of concept” meaning no further information is likely to be gained by continuing interviews in the same population.

Descriptive statistics were generated for demographic and concept rating data. Information for the cognitive portion of the interviews was collected into a summary grid that organized and related the response from each participant to the item that was queried. Difficulties in understanding the item content were also noted in the cognitive summary grid.

## Results

### Population characteristics

Of the 20 participants interviewed in the study, the mean age was 57.4 years and 90% were women (Table 1). Most (90%) participants were white and 10% were Hispanic. Fourteen were in the US (Seattle, WA; Austin, TX; El Paso, TX; Lansing, MI; Grand Rapids, MI; New York City, NY; and San Francisco, CA) and 6 were in Canada (Toronto, ON; Victoria, BC; and Vancouver, BC). The majority (55%) were employed full-time, whereas 30% were retired. The most common comorbidities were allergies (50%) and arthritis (50%), and the mean severity of worst itch based on a 101-point NRS was 89.3 (a score of 100 represents the “Worst Itching You Can Imagine” and 0 represents “NO Itching at All”).

**Table 1:** Participant demographic and disease characteristics

Characteristic	*N* = 20
Age, y	
Mean (SD)	57.4 (11.2)
Range	32–74
Female, *n* (%)	18 (90)
Race, *n* (%)	
White	18 (90)
Hispanic	2 (10)
Highest level of education completed, *n* (%)	
High school	1 (5)
Some college	7 (35)
Bachelor’s degree	6 (30)
Graduate or professional school	6 (30)
Current employment status, *n* (%)	
Full time	11 (55)
Part time	1 (5)
Unemployed for > 1 year	1 (5)
Retired	6 (30)
Unable to work because of PBC, CPS/fibromyalgia	1 (5)
Household income, US $, *n* (%)	
$15,000–$24,999	2 (10)
$25,000–$34,999	1 (5)
$35,000–$49,999	2 (10)
$50,000–$74,999	6 (30)
$75,000–$99,999	2 (10)
$100,000–$124,999	1 (5)
≥$125,000	5 (25)
Declined to answer	1 (5)
Comorbidities, *n* (%)^a^	
Allergies (hay fever, chronic sinus trouble, other)	10 (50)
Arthritis or rheumatism	10 (50)
Asthma or other severe lung problems^b^	4 (20)
Back problems (including disc or spine)	7 (35)
Depression	2 (10)
Diabetes or high blood sugar	3 (15)
Headache or migraine	3 (15)
Heart trouble^c^	1 (5)
High blood pressure or hypertension	7 (35)
Nervousness or anxiety disorder	2 (10)
Thyroid problems	1 (5)
Trouble seeing (even with glasses or contact lenses)	4 (20)
Itch NRS (scale 0–100)^d^	
Severity of worst itch, mean (SD)	89.3 (14.1)
Severity of usual amount of itch, mean (SD)	50.5 (24.5)

*CPS*, chronic pain syndromes; *NRS* numerical response scale; *PBC* primary biliary cholangitis; *SD*, standard deviation; *US*, United States

^a^One participant each (5%) reported celiac disease, chronic reflux, fibromyalgia, hepatitis C, mixed nonspecific autoimmune disease, osteoarthritis, osteoporosis, polyps, Raynaud’s disease, Sjogren’s syndrome, or sphincter of Oddi dysfunction

^b^Chronic bronchitis, chronic obstructive pulmonary disorder, or emphysema

^c^Angina, congestive heart failure, coronary artery disease, use of cardiac pacemaker

^d^During the concept elicitation portion of the interview, participants were asked to complete a series of rating exercises using a NRS, with the 0 end of the scale representing “NO Itching at All,” and 100 representing “Worst Itching You Can Imagine”

Sixteen patients signed the optional Medical Information Release Form authorizing the study team to request limited medical information from their medical provider. Of the 16 forms sent to providers, 10 were returned. Four additional patients brought their own medical records to the interviews. Therefore, additional limited medical information from medical providers was available for 14 participants. PBC diagnosis was confirmed in all 14 patients, with a mean (standard deviation) duration of disease of 9.6 (6.0) years. Twelve of these 14 participants (86%) had received ursodeoxycholic acid (past or present) to treat their PBC. Only 4 reported they were currently using medication to relieve their itching.

Patients who were screened but did not fully meet the eligibility criteria were considered on an individual basis to determine whether the particular criteria deviation might lead to confounding or conflict with the study design or objectives. Four of these subjects were authorized to participate despite not meeting one or more eligibility criteria, one subject cancelled the interview and the three remaining subjects were interviewed and included in the analysis. Of those included, 1 patient reported average itching of level 3 (just below the threshold of 4), although this patient reported having itching above a 4 at times during the recall period; 1 patient had been diagnosed with hepatitis C, but had been declared cured after treatment for the infection; and 1 patient reported infrequent minor flare-ups of eczema but was not experiencing eczema during screening or the interview for the current study.

### Concept elicitation (CE) interviews

Saturation of concept (the point where no new concepts emerged) for the Itch Diary was reached by the end of the third transcript group (i.e., 4 groups with 5 transcripts each). Inter-rater agreement in assignment of codes between the 2 raters was high at 94% and 95% for the 2 dual-coded transcripts.

The CE portion of the interviews confirmed relevance of the concepts presented in the items of the PBC-40 and Itch Diary (both AM and PM versions). Itching-related signs and symptoms were reported by all patients, and was the most commonly expressed symptom subdomain when assessed by symptom concept code frequency (46% of all symptom expressions) followed by energy-related concepts (14% of total symptoms expressed) and additional signs and symptoms (13% of total symptoms expressed) (Table 2). Specific codes in terms of how patients described the term and additional descriptors are reported in Table 2.

**Table 2:** Summary of symptom concept code frequency totals by subdomain

PBC Symptom Subdomains and Concepts	Total Language Expressions Within Concept, *n* (%) *N* = 642	Transcripts Contributing to Concept Expressions, *n* (%) *N* = 20
Itching-related signs and symptoms	293 (46)	20 (100)
Itching	254 (39.6)	20 (100)
Hives	12 (1.9)	3 (15)
Burning	9 (1.4)	3 (15)
Rash	9 (1.4)	3 (15)
Tingling	8 (1.2)	4 (20)
Dry patches	1 (0.2)	1 (5)
Energy-related signs and symptoms	87 (14)	18 (90)
Fatigue	44 (6.9)	17 (85)
Tiredness	16 (2.5)	9 (45)
Low energy	14 (2.2)	6 (30)
Exhaustion	8 (1.2)	7 (35)
Weakness	5 (0.8)	4 (20)
Additional signs and symptoms	86 (13)	19 (95)
Other symptoms^a^	20 (3.1)	11 (55)
Dry eyes	13 (2.0)	10 (50)
Gallstones	9 (1.4)	8 (40)
Dry mouth	8 (1.2)	8 (40)
Weak/brittle bones	8 (1.2)	5 (25)
Fat deposits on skin	8 (1.2)	6 (30)
Darkening of skin	8 (1.2)	6 (30)
Hair loss	3 (0.5)	2 (10)
Jaundice	3 (0.5)	2 (10)
Sleep disturbance	3 (0.5)	3 (15)
Swelling	3 (0.5)	3 (15)
Pain and discomfort	77 (12)	19 (95)
Joint pain/aches	23 (3.6)	10 (50)
Discomfort in right side	21 (3.3)	11 (55)
Muscle pain/aches	9 (1.4)	5 (25)
General pain	8 (1.2)	6 (30)
Other pain and discomfort	8 (1.2)	8 (40)
Abdominal pain	4 (0.6)	4 (20)
Bone pain/aches	4 (0.6)	3 (15)
GI signs and symptoms	69 (11)	16 (80)
Diarrhea	17 (2.6)	10 (50)
Nausea	12 (1.9)	7 (35)
Abdominal bloating	11 (1.7)	10 (50)
Other GI symptoms	10 (1.6)	7 (35)
Urgency with bowel movements	7 (1.1)	7 (35)
Greasy diarrhea	6 (0.9)	5 (25)
Indigestion/heartburn	3 (0.5)	3 (15)
Changes in appetite	3 (0.5)	3 (15)
Cognitive signs and symptoms	30 (5)	10 (50)
Difficulty concentrating	11 (1.7)	7 (35)
Difficulty remembering things	9 (1.4)	7 (35)
Other cognitive symptoms	4 (0.6)	2 (10)
Brain fog	3 (0.5)	2 (10)
Confusion	3 (0.5)	2 (10)

*GI*, gastrointestinal; *PBC*, primary biliary cholangitis

^a^Other symptoms include: bruises easily, cellulitis, changes in taste, chest infections, eye problems, fibromatosis, flushed, fluid in abdomen, heat intolerance, kidney stones, no hair growth, redness, restless legs, shingles, shortness of breath, and sweating

During the CE portion of the interview, participants were given the opportunity to spontaneously report symptoms related to their PBC before follow-up probing questions were asked. The symptom concepts most often mentioned spontaneously were “fatigue” (75%; 15/20 participants) and “itching on the legs” (75%; 15/20 participants). Other common spontaneously reported symptom concepts were “itching on the arms” (55%; 11/20 participants), “itching on the side” (45%; 9/20 participants), and “itching on the back” (45%; 9/20 participants). Follow-up probing questions did not notably increase the reports related to itching, although the number of participants reporting weakness, gastrointestinal signs and symptoms, cognitive signs and symptoms, and pain and discomfort were markedly increased (Table 3).

**Table 3:** Spontaneous vs probed symptom expressions during the CE portion of the interviews

Symptom Reported, *n* (%)	Participants, *N* = 20			
	Spontaneous	Probed	Not Affected	Not Reported
Itching-related signs and symptoms				
Itching on the legs	15 (75)	–	–	5 (25)
Itching on the arms	11 (55)	2 (10)	–	7 (35)
Itching on the back	9 (45)	1 (5)	–	10 (50)
Itching on the sides	9 (45)	1 (5)	–	10 (50)
Itching on the abdomen	7 (35)	2 (10)	–	11 (55)
Itching on the head	6 (30)	–	–	14 (70)
Itching on the feet	3 (15)	–	–	17 (85)
Itching on the hands	3 (15)	–	–	17 (85)
Itching on the face	2 (10)	1 (5)	–	17 (85)
Itching on the chest	1 (5)	–	–	19 (95)
Itching on the groin	1 (5)	–	–	19 (95)
Hives	1 (5)	–	–	19 (95)
Rash	1 (5)	–	–	19 (95)
Energy-related signs and symptoms				
Fatigue	15 (75)	1 (5)	2 (10)	2 (10)
Tiredness	6 (30)	–	–	14 (70)
Lack of energy	3 (15)	–	–	17 (85)
Exhausted	2 (10)	–	–	18 (90)
Weakness	2 (10)	10 (50)	8 (40)	–
Gastrointestinal signs and symptoms				
Nausea	6 (30)	–	–	14 (70)
Diarrhea	4 (20)	8 (40)	8 (40)	–
Other^a^	3 (15)	1 (5)	–	16 (80)
Abdominal bloating	2 (10)	10 (50)	6 (30)	2 (10)
Changes in appetite	–	5 (25)	14 (70)	1 (5)
Greasy diarrhea	1 (5)	7 (35)	11 (55)	1 (5)
Urgency with bowel movements	–	12 (60)	7 (35)	1 (5)
Cognitive signs and symptoms				
Difficulty concentrating	3 (15)	11 (55)	6 (30)	–
Difficulty remembering things	2 (10)	12 (60)	6 (30)	–
Pain and discomfort				
Bone pain/aches	5 (25)	2 (10)	–	13 (65)
Muscle pain/aches	4 (20)	–	–	16 (80)
Abdominal pain	3 (15)	9 (45)	7 (35)	1 (5)
Discomfort in right side	3 (15)	11 (55)	5 (25)	1 (5)
General pain	–	4 (20)	4 (20)	12 (60)
Joint pain/aches	3 (15)	2 (10)	–	15 (75)
Other^b^	1 (5)	1 (5)	–	18 (90)
Additional signs and symptoms				
Dry eyes	6 (30)	8 (40)	6 (30)	–
Difficulty staying asleep	5 (25)	11 (55)	4 (20)	–
Dry mouth	4 (20)	9 (45)	7 (35)	–
Hair loss	4 (20)	–	–	16 (80)
Other^c^	4 (20)	–	–	16 (80)
Difficulty falling asleep	2 (10)	8 (40)	9 (45)	1 (5)
Weak/brittle bones	2 (10)	1 (5)	17 (85)	–
Darkening of the skin	1 (5)	7 (35)	12 (60)	–
Fatty deposits on skin	1 (5)	5 (25)	14 (70)	–
Gall stones	–	3 (15)	3 (15)	14 (70)
Jaundice	1 (5)	1 (5)	18 (90)	–
Kidney stones	–	5 (25)	9 (45)	6 (30)
Swelling	–	10 (50)	10 (50)	–

*CE*, concept elicitation

^a^Acid reflux, constipation, fewer stools, don’t process vitamins/minerals properly

^b^Headache, migraine

^c^Weight gain, weight loss, restless legs, sweating, skin flushed, heavy menstruation

During the interview, participants were asked to refer to the symptoms they had described and identify the top 1 or 2 symptoms that were the worst for them. The symptoms most commonly chosen were itching (worst in 40% of participants, second worst in 25% of participants) and fatigue (worst in 35% of participants, second worst in 35% of participants). Participants identified the most common location for itching as legs (75%), followed by arms (65%), torso (60%), and feet (50%).

A series of questions was asked during the CE portion of the interviews to explore the impact of PBC on patients’ lives and the language used by participants to describe these impacts. Changes in daily performance was the most commonly expressed impact subdomain when assessed by impact concept code frequency (21% of total impact expressions) followed by emotional functioning (18% of total impact expressions) and sleep difficulties (17% of total impact expressions) (Table 4).

**Table 4:** Summary of impact concept code frequency totals by subdomain

PBC Impact Subdomains and Concepts	Total Participant Symptom Expressions Within Concept, *n* (%) *N* = 571	Transcripts Contributing to Concept Expressions, *n* (%) *N* = 20
Changes in daily performance	119 (21)	18 (90)
Emotional functioning	103 (18)	19 (95)
Sleep difficulties caused by itching or other symptoms	98 (17)	18 (90)
Limitations to relationships and social functioning	87 (15)	16 (80)
Limitations to lifestyle and activities	84 (15)	20 (100)
Additional impacts^a^	80 (14)	19 (95)

*PBC*, primary biliary cholangitis

^a^Decreased quality of life, economic burden, scratching until skin is raw or infected, treatment burden, physical discomfort, and weight gain

Within these subdomains, the most frequently cited concepts overall were “scratching until skin is raw/infected” (38 expressions by 16 participants [80%]) in the additional impacts subdomain, “difficulty staying asleep” (38 expressions by 16 participants [60%]) in the sleep difficulties subdomain, “social activities limited” (37 expressions by 14 participants [70%]) in the limitations to relationships and social functioning subdomain, “limitations at work” (34 expressions by 15 participants [75%]) in the changes to daily performance subdomain, and “needing to change their diet” (32 expressions by 15 participants [75%]) in the limitations to lifestyle and activities subdomain.

Similar to the symptom concepts, participants were allowed to spontaneously report impact concepts related to their PBC before follow-up probing questions were asked. The impact concepts most often mentioned spontaneously were “limitations at work” (65%; 13/20 participants) and “difficulty staying asleep” (65%; 13/20 participants). Other common spontaneously reported impact concepts were “needing to change their diet” (60%; 12/20 participants) and “scratching until skin is raw/infected” (55%; 11/20 participants). Follow-up probing questions increased the number of participants reporting most of the impact concepts (Table 5).

**Table 5:** Spontaneous vs probed impact expressions during the CE portion of the interviews

Symptom Reported, *n* (%)	Participants, *N* = 20			
	Spontaneous	Probed	Not Affected	Not Reported
Emotional functioning				
Anxiety	7 (35)	2 (10)	3 (15)	8 (40)
Embarrassment	5 (25)	1 (5)	3 (15)	11 (55)
Depression	4 (20)	1 (5)	3 (15)	12 (60)
Worry	3 (15)	5 (25)	3 (15)	9 (45)
Anger	2 (10)	2 (10)	3 (15)	13 (65)
Frustration	2 (10)	5 (25)	3 (15)	10 (50)
Stress	2 (10)	4 (20)	3 (15)	11 (55)
Guilt	1 (5)	3 (15)	3 (15)	13 (65)
Other^a^	1 (5)	–	–	19 (95)
Changes in daily performance				
Work	13 (65)	2 (10)	3 (15)	2 (10)
General functioning/daily routine	4 (20)	5 (25)	3 (15)	8 (40)
Housework/chores	4 (20)	5 (25)	3 (15)	8 (40)
Needs to pace self	3 (15)	11 (55)	6 (30)	–
Takes longer than usual to recover	3 (15)	14 (70)	2 (10)	1 (5)
Takes longer than usual to complete tasks	2 (10)	13 (65)	5 (25)	–
Need to force self	1 (5)	12 (60)	7 (35)	–
Other^b^	1 (5)	–	3 (15)	16 (80)
Limitations to lifestyle and activities				
Changes to diet	12 (60)	3 (15)	4 (20)	1 (5)
Changes to alcohol consumption	7 (35)	6 (30)	6 (30)	1 (5)
Exercise/sports	5 (25)	5 (25)	6 (30)	4 (20)
Travel	5 (25)	2 (10)	6 (30)	7 (35)
Unable to plan things in advance	4 (20)	6 (30)	9 (45)	1 (5)
Clothing restrictions	2 (10)	–	–	18 (90)
Other^c^	–	1 (5)	–	19 (95)
Limitations to social functioning				
Social activities limited	8 (40)	6 (30)	6 (30)	–
Family relations affected	6 (30)	4 (20)	9 (45)	1 (5)
Friends relations affected	5 (25)	2 (10)	8 (40)	5 (25)
Relationships with partner affected	3 (15)	3 (15)	8 (40)	6 (30)
Workplace relations affected	2 (10)	2 (10)	8 (40)	8 (40)
Other^d^	1 (5)	–	–	19 (95)
Less interest in sex	–	5 (25)	14 (70)	1 (5)
Sexual function issues	–	3 (15)	14 (70)	3 (15)
Sleep difficulties caused by itching or other symptoms				
Difficulty staying asleep	13 (65)	3 (15)	4 (20)	–
Difficulty falling asleep	7 (35)	–	4 (20)	9 (45)
Needs to take naps	3 (15)	7 (35)	10 (50)	–
Reduced sleep quality	3 (15)	1 (5)	4 (20)	12 (60)
Wakes up too early	3 (15)	2 (10)	4 (20)	11 (55)
Goes to bed early	–	10 (50)	10 (50)	–
Additional impacts				
Scratching until skin is raw/infected	11 (55)	7 (35)	2 (10)	–
Decreased quality of life	3 (15)	12 (60)	5 (25)	–
Economic burden	2 (10)	6 (30)	11 (55)	1 (5)
Treatment burden	2 (10)	1 (5)	–	17 (85)

*CE*, concept elicitation

^a^Concern about having children

^b^School

^c^Gardening

^d^Social stigma

Participants were also asked to choose 1 to 2 impacts that were the worst for them. Responses varied widely, but 3 participants (15%) selected “general function/daily routine” and 2 participants (10%) selected “reduced sleep quality.”

### Cognitive interviews and PRO revisions

The cognitive portion of the interviews resulted in several changes to the Itch Diary and PBC-40 (Table 6). During wave 1, 2 items were dropped from the Itch Diary (1 from the AM and 1 from the PM diary) because participants felt that these were very similar to other items. The question stems were changed in 6 items (1 in the AM and 5 in the PM diary) to make the items clearer and easier to answer. The response scale was changed from an NRS version to a verbal response scale (VRS) version in 1 item in the AM Itch Diary and 7 items in the PM Itch Diary to reduce confusion in interpretation of answers. For example, many of the questions asked “how much time…” and some participants interpreted the number options as percentages, whereas others interpreted the options as number of hours. Changing the response to a VRS helped to more clearly and consistently define each of the response options (i.e., “none of the time,” “a little of the time,” etc).

**Table 6:** Summary of changes to Itch Diary and PBC-40 as a result of cognitive interviews

Measure	Total Items in Final Measure	Items Left Unchanged	Items Removed	Items Added	Items Revised, n, Description
AM Itch Diary	7	4	1	1	2 Item wording revised – 1 Response scale revised – 1
PM Itch Diary	11	1	1	2	8 Item wording revised – 5 Response scale revised – 7
PBC-40	43	35	0	0	8 Item wording revised – 7 Response scale revised – 1

*AM*, morning; *PBC*, primary biliary cholangitis; *PM*, evening

Further revisions were made to question stems and the response scale after wave 2. These changes included additional wording changes to 1 of the items in the AM and PM diaries, as well as changes to the response options of these diaries; additionally, the NRS versions of select items were added back into the measure for use in an upcoming clinical trial for testing against the VRS versions.

Three of the item revisions in the PBC-40 during wave 1 were to adapt from British English in the original version to terms more common in North America (i.e., “holiday” vs “vacation”). During wave 2, additional revisions were made to 3 items to further clarify their meaning. For example, 1 item asked about aching in the long bones of the patient’s arms and legs. Some participants had trouble responding because they experienced aching in the arms, but not in the legs or vice versa. Therefore, the stem was changed to “arms or legs” to make it clearer that participants could respond if they had aching in only one location.

There were no major issues or revisions made to the PGIS-Itch, PGIC-Itch, or 5-D Itch measures; the PGIS-Itch and PGIC-Itch were determined to be appropriate anchor measures.

During wave 1, the response options were revised for 1 item (“always” changed to “almost always” with regard to how frequently the participant had diarrhea) in the Weekly Gastrointestinal Diary. No further revisions were made to this questionnaire during wave 2.

### Time to complete PRO measures

The mean (range) time to complete the PRO measures during wave 1 of the interviews was 57.4 (38–111) seconds for the AM Itch Diary, 88 (39–100) seconds for the PM Itch Diary, and 252.8 (167–407) seconds (4.2 [2.8–6.8] minutes) for the PBC-40. The mean (range) time to complete the PRO measures during wave 2 of the interviews was 46.5 (28–75) seconds for the AM Itch Diary, 89.8 (47–120) seconds for the PM Itch Diary, and 291.9 (161–420) seconds (4.9 [2.7–7.0] minutes) for the PBC-40.

## Discussion

Qualitative interviews of 20 North American participants with PBC confirmed the overall content of the Itch Diary and weekly PBC-40 measures in terms of symptoms and disease impact. Qualitative patient interviews are recognized as being an integral part of developing quality of life measures and are recommended by the FDA for the development of PRO measures to support label claims [15, 16]. Itching-related symptoms were the most commonly expressed symptom concept both spontaneously and overall. Patients reported that itching and fatigue were the worst of their PBC symptoms. Scratching, sleep issues, and work limitations associated with PBC appeared to have the most important impacts on daily life.

While scratching is a behavior that can be directly observed by others, and is an activity that leaves visible evidence by way of marks and bleeding, itching is a symptom and can only be described by the individual experiencing this sensation. As demonstrated in the current study and others, PBC-related itching can be burdensome for patients [6, 7]. However, previously available tools to assess itch were simplistic measures of worst itch or were not disease specific, or had limited evidence to support their content validity [8]. The Itch Diary was developed to meet the need for a PRO specifically designed to measure the impact of PBC-related itch. Our goal in this study was to confirm the content validity of the daily Itch Diary, which was the primary endpoint. The Itch Diary is independent from the PBC-40 questionnaire, and we wanted to ensure that the diary captured items relevant to patients with pruritus due to PBC. The findings reported here suggest that the Itch Diary will be a useful outcome tool when assessing the efficacy of new treatments for PBC-related itching.

In addition to the Itch Diary, the current study assessed an adapted version of the PBC-40 using a recall period of “past 7 days” instead of “last 4 weeks.” Although the cognitive interviews identified minor issues in the PBC-40, any major changes needed to be discussed and approved by the original developers. After consulting with the original developers, revisions were mainly limited to making minor grammatical changes, such as changing terms mainly used in British English to terms more commonly used and understandable to North Americans.

A limitation of this study is that eligibility was based on self-reported diagnosis of PBC. However, of the 14 participants with additional medical information provided by their physician, all 14 had confirmed PBC. Another limitation is that the patient population was enriched for patients with moderate to severe PBC-related itch. Thus, the applicability of the Itch Diary to the generalized PBC population, or in other conditions, is unknown at this time.

## Conclusion

Qualitative interviews confirmed the relevance and comprehension of the Itch Diary and the weekly version of the PBC-40 as fit-for-purpose measures to assess the relevant symptoms and impacts associated with PBC and PBC-related itching, and to support the content validity of the measures. Adaptation of the PBC-40 to a weekly recall period and for North American English was successful. These measures will be useful in evaluating the efficacy of new treatments for PBC-related itch, although further work is needed to fully evaluate the psychometric properties of these measures.

## Abbreviations

CE: concept elicitation
FDA: US Food and Drug Administration
ID: Itch Diary
NRS: numerical response scale
PBC: primary biliary cholangitis
PGIC-Itch: Patient Global Impression of Change for Itch
PGIS-Itch: Patient Global Impression of Severity for Itch; PRO: patient-reported outcome
VAS: Visual Analogue Scale; VRS: verbal response scale

## References

[1] Hirschfield, G. M., Dyson, J. K., Alexander, G. J. M., Chapman, M. H., Collier, J., Hubscher, S., et al. (2018). The British Society of Gastroenterology/UK-PBC primary biliary cholangitis treatment and management guidelines. *Gut*. 10.1136/gutjnl-2017-315259. 10.1136/gutjnl-2017-315259 PMC6109281 29593060

[2] Tsuneyama K, Baba H, Morimoto Y, Tsunematsu T, Ogawa H (2017). Primary biliary cholangitis: Its pathological characteristics and immunopathological mechanisms. J Med Investig.

[3] Lu, M., Zhou, Y., Haller, I.V., Romanelli, R. J., VanWormer, J. J., Rodriguez, C. V., et al. (2017). Increasing prevalence of primary biliary cholangitis and reduced mortality with treatment. *Clinical Gastroenterology and Hepatology: The Official Clinical Practice Journal of the American Gastroenterological Association*. doi: 10.1016/j.cgh.2017.12.033. 10.1016/j.cgh.2017.12.033 29277621

[4] Myers RP, Shaheen AA, Fong A, Burak KW, Wan A, Swain MG (2009). Epidemiology and natural history of primary biliary cirrhosis in a Canadian health region: A population-based study. Hepatology (Baltimore, MD).

[5] Talwalkar JA, Souto E, Jorgensen RA, Lindor KD (2003). Natural history of pruritus in primary biliary cirrhosis. Clinical Gastroenterology and Hepatology: The Official Clinical Practice Journal of the American Gastroenterological Association.

[6] Mells GF, Pells G, Newton JL, Bathgate AJ, Buroughs AK, Heneghan MA (2013). Impact of primary biliary cirrhosis on perceived quality of life: The UK-PBC national study. Hepatology (Baltimore, MD).

[7] Rishe E, Azarm A, Bergasa NV (2008). Itch in primary biliary cirrhosis: A patients’ perspective. Acta Derm Venereol.

[8] Hegade VS, Bolier R, Oude Elferink RP, Beuers U, Kendrick S, Jones DE (2016). A systematic approach to the management of cholestatic pruritus in primary biliary cirrhosis. Frontline Gastroenterology.

[9] Elman S, Hynan LS, Gabriel V, Mayo MJ (2010). The 5-D itch scale: A new measure of pruritus. Br J Dermatol.

[10] Jacoby A, Rannard A, Buck D, Bhala N, Newton JL, James OF (2005). Development, validation, and evaluation of the PBC-40, a disease specific health related quality of life measure for primary biliary cirrhosis. Gut.

[11] Gilchrist, K., & Vallow, S. (2015). Development of electronic diary in patients with primary biliary cirrhosis. *Value Health*. 10.1016/j.jval.2015.03.183.

[12] Hegade VS, Kendrick SF, Dobbins RL, Miller SR, Richards D, Storey J (2016). BAT117213: Ileal bile acid transporter (IBAT) inhibition as a treatment for pruritus in primary biliary cirrhosis: Study protocol for a randomised controlled trial. BMC Gastroenterol.

[13] Hegade VS, Kendrick SF, Dobbins RL, Miller SR, Thompson D, Richards D (2017). Effect of ileal bile acid transporter inhibitor GSK2330672 on pruritus in primary biliary cholangitis: A double-blind, randomised, placebo-controlled, crossover, phase 2a study. Lancet (London, England).

[14] Houts, C. R., Morlock, R., Blum, S.I., Edwards, M.C., & Wirth, R. J. (2018). Scale development with small samples: A new application of longitudinal item response theory. Qual Life Res*:* An International Journal of Quality of Life Aspects of Treatment, Care and Rehabilitation. 10.1007/s11136-018-1801-z. 10.1007/s11136-018-1801-z 29423756

[15] US Department of Health and Human Services FDA Center for Drug Evaluation and Research; US Department of Health and Human Services FDA Center for Biologics Evaluation and Research; US Department of Health and Human Services FDA Center for Devices and Radiological Health (2006). Guidance for industry: Patient-reported outcome measures: Use in medical product development to support labeling claims: Draft guidance. Health Qual Life Outcomes.

[16] Guyatt GH, Cook DJ (1994). Health status, quality of life. and the individual JAMA.

